# Reframing obesity-related HFpEF as a multiorgan syndrome: incretin-based therapies and imaging endpoints

**DOI:** 10.1007/s10741-026-10629-z

**Published:** 2026-04-13

**Authors:** Masliza Mahmod, Andrea Dennis, Jie Lian, Caitlin Langford, Kazem Rahimi, Malgorzata Wamil

**Affiliations:** 1 Division of Cardiovascular Medicine, Radcliffe Department of Medicine, University of Oxford, Oxford, UK; 2 Perspectum Ltd, Oxford, UK; 3 Deep Medicine, Nuffield Department of Women’s & Reproductive Health, University of Oxford, Oxford, UK; 4 Cardiology Department, Great Western Hospital NHS Trust, Swindon, UK; 5 Mayo Clinic Healthcare, London, UK

**Keywords:** GLP-1 receptor agonists, Dual incretin agonists, Heart failure with preserved ejection fraction (HFpEF), Obesity, Multiorgan imaging, Magnetic resonance imaging (MRI)

## Abstract

Obesity-related heart failure with preserved ejection fraction (HFpEF) is increasingly recognised as a multiorgan cardio–renal–hepatic–metabolic (CRHM) syndrome. Obesity promotes HFpEF through metabolic dysfunction, systemic inflammation, haemodynamic overload and ectopic adiposity. Increased visceral and epicardial fat has been linked with downstream involvement of the heart, liver and kidneys, resulting in a distinct phenotype with high symptom burden and functional limitation. Incretin based therapies such as glucagon-like peptide-1 receptor agonist (GLP-1 RA) are growing in popularity in CRHM conditions owing to positive effects on glycaemia, weight loss and systemic inflammation, leading to reduced adverse clinical outcomes. In obesity-related HFpEF, emerging data suggest GLP-1 RA and glucose-dependent insulinotropic polypeptide (GIP)/GLP-1 agonists (also called incretin-based therapies) improve symptoms, exercise capacity, congestion, and reduce heart failure events, while also leading to reduced ectopic fat depositions in multiple organs and favourable cardiac remodelling. Incretin-based, weight-directed therapies therefore represent a promising strategy for a therapeutically challenging HFpEF phenotype. Future clinical trials incorporating multiorgan imaging endpoints across the heart, liver, adipose tissue and kidneys are needed to clarify the mechanisms of benefit and to better define the role of weight-reduction therapies in obesity-related HFpEF.

## Obesity-related HFpEF: a multiorgan perspective

Obesity is a major driver of cardiometabolic disease and has contributed to the rising prevalence of heart failure with preserved ejection fraction (HFpEF) [1–3]. An obesity-related HFpEF phenotype is increasingly recognised, characterised by congestion, exercise intolerance and high symptom burden often in the context of type 2 diabetes, hypertension, dyslipidaemia and obstructive sleep apnoea [4, 5].

Clinically, HFpEF is characterised by disproportionate exertional dyspnoea, exercise intolerance, systemic congestion and a high comorbidity burden, and these features are often accentuated in patients with obesity [4, 6]. Visceral and ectopic fat accumulation, skeletal muscle fat infiltration, and increased pericardial restraint have all been linked in observational and mechanistic studies to reduced cardiopulmonary and peripheral reserve. This may explain why symptoms and congestion are particularly prominent in obesity-related HFpEF, supporting the view that this phenotype behaves as a systemic cardiometabolic syndrome rather than a purely cardiac disorder [4, 7].

Obesity promotes HFpEF through combined haemodynamic and metabolic mechanisms. The haemodynamic mechanisms include expansion of adipose tissue, increasing circulating volume and cardiac output, leading to chronic elevations in preload and afterload. Over time, these favour left ventricular hypertrophy, reduced chamber distensibility and elevated filling pressures [4, 5]. These haemodynamic changes are particularly important because they create the physiological substrate for the development of symptoms and congestion. Although patients with the obese HFpEF phenotype often have higher left-sided filling pressures, especially during exercise, pulmonary congestion typically occurs at substantially higher wedge pressures and in the presence of additional haemodynamic abnormalities; thus, elevated filling pressures are necessary but not sufficient for acute decompensation [6]. Central obesity further increases intra-abdominal and intrathoracic pressures, limiting lung expansion and venous return and worsening exertional dyspnoea [5].

The metabolic mechanism of obese HFpEF is primarily related to increased fat depots both in and surrounding the vital organs. Visceral adiposity is the fat stored within the abdomen and is a defining feature of cardiometabolic disease. Ectopic fat refers to fat stored within the organs. In the heart, epicardial adipose tissue (EAT) is a metabolically active visceral fat depot between the myocardium and visceral pericardium [8, 9]. It is contiguous with the myocardium and coronary arteries and acts as a local source of free fatty acids and pro-inflammatory mediators [9]. Increased EAT volume is linked to coronary atherosclerosis, atrial fibrillation and HF [8, 9]. In HFpEF, a higher EAT burden may be associated with worse diastolic function, higher filling pressures, and greater congestion [4, 9]. Pericardial adipose tissue (PAT), located external to the fibrous pericardium, is less tightly coupled to the myocardium but correlates with systemic inflammation and cardiometabolic risk [8, 9]. Together, EAT and PAT, in the context of visceral adiposity, are thought to contribute to concentric remodelling, augment pericardial restraint, and may increase susceptibility to elevated filing pressures and pulmonary congestion, although these links are largely based on observational and mechanistic data [5].

Obesity-related HFpEF rarely occurs in isolation. Visceral adiposity is a common feature of HFpEF as well as metabolic dysfunction–associated steatotic liver disease (MASLD), chronic kidney disease and skeletal muscle dysfunction [1, 2]. Within the heart, the obesity-related HFpEF phenotype is characterised by concentric LV remodelling, left atrial enlargement and dysfunction, and impaired pulmonary vascular reserve, features that may contribute to exertional intolerance and pulmonary congestion despite preserved ejection fraction [4]. Many of these conditions occur concurrently, whilst each will have its own specific mechanism that leads to poor quality of life. For instance, in MASLD, hepatic steatosis results in chronic inflammation and subsequent fibrosis [10]. Peri-renal fat is closely related to chronic kidney disease (CKD) [11], which results in progressive loss of estimated glomerular filtration rate, reinforcing neurohumoral activation and sodium retention [5]. Obesity related muscle fat infiltration is a major cause of sarcopenia, which is characterised by loss of muscle strength and functions caused by microvascular abnormalities that limit oxygen delivery and utilisation in skeletal muscles [12, 13]. These extracardiac changes contribute to exertional intolerance, fluid retention and poor quality of life. Obesity-related HFpEF is therefore best viewed as a multiorgan cardio–renal–hepatic–metabolic syndrome (CRHM) rather than a purely cardiac disorder [4, 5]. This multiorgan construct also inherently favours imaging-based phenotyping, because modern multimodality imaging can quantify cardiac, hepatic, adipose and renal involvement in parallel.

Glucagon-like peptide-1 receptor agonist (GLP-1 RA) therapy and dual incretin agonists provide a mechanistically attractive approach for obesity-related HFpEF. GLP-1 RAs reduce appetite and energy intake, slow gastric emptying and improve glycaemic control, blood pressure and atherogenic lipids, leading to sustained weight loss and lower haemodynamic and metabolic load [14–17]. Dual incretin agonists, which also activate the glucose-dependent insulinotropic polypeptide receptor, achieve greater weight loss and larger reductions in visceral fat and inflammatory markers than GLP-1 RA monotherapy [17, 18]. Beyond weight loss and glycaemic control, incretin-based therapies reduce ectopic fat (particularly liver and epicardial/pericardial depots), improve markers of steatohepatitis, and slow chronic kidney disease progression, supporting their potential as multiorgan treatments in obesity-related HFpEF [18–20].

From the available evidence, a pragmatic way to organise reported multiorgan effects is to distinguish three partially overlapping domains, each assessable with prespecified imaging and biomarker endpoints in clinical trials. First, weight-dependent effects reflect total mass reduction and are expected to improve functional capacity and haemodynamic load, for example, improvements in 6-minute walk distance and Kansas City Cardiomyopathy Questionnaire clinical summary score (KCCQ-CSS), reductions in congestion and haemodynamic surrogates, and lower blood pressure. Second, changes in adipose tissue distribution and ectopic fat are best captured as changes in visceral and organ fat depots, including reductions in visceral adipose tissue, epicardial and hepatic fat, skeletal muscle fat infiltration, and liver injury or fibrosis surrogates. Third, potentially weight-independent pathways include anti-inflammatory and endothelial mechanisms and renal sodium-handling effects, which may contribute to haemodynamic unloading beyond weight loss, with trial endpoints such as reductions in hsCRP and other inflammatory markers, improvements in vascular function surrogates, lower albuminuria and renal injury biomarkers, and modest blood-pressure lowering. Framing obesity-related HFpEF in this way supports the design of adequately powered RCTs with prespecified multiorgan endpoints to disentangle mechanisms and attribute benefits beyond weight loss alone.

## HFpEF-specific trials of incretin-based therapies

Major GLP-1 RA and GIP/GLP-1 dual agonist trials across obesity and obesity-associated HFpEF are summarised in Table 1, including both cardiovascular outcome trials and dedicated HFpEF–obesity studies.

**Table 1:** Major GLP-1 receptor agonist and dual incretin trials in obesity and obesity-related HFpEF

Trial	Population	Intervention vs. placebo	Key findings
**Non-HFpEF CV-obesity trials**			
LEADER [21]	T2D with established CVD or high CV risk	Liraglutide 1.8 mg once daily vs. placebo	13% relative reduction in MACE; HF hospitalisation neutral; established GLP-1 RA as cardioprotective beyond glucose lowering
SUSTAIN-6 [22]	T2D with high CV risk	Semaglutide 0.5/1.0 mg once weekly vs. placebo	26% relative reduction in MACE; HF outcomes exploratory/neutral; supports CV benefit of semaglutide in high-risk T2D
REWIND [23]	Broad T2D population (majority without established CVD)	Dulaglutide 1.5 mg once weekly vs. placebo	12% relative reduction in MACE in a broad T2D population; HF hospitalisations not significantly reduced but no safety concern
STEP-1 [17]	Overweight/obesity without diabetes	Semaglutide 2.4 mg once weekly vs. placebo + lifestyle	~ 15% mean weight loss; reduced waist circumference and hsCRP; provides weight-loss rationale for semaglutide in obesity
SELECT [16]	Overweight/obesity with established atherosclerotic CVD, no diabetes	Semaglutide 2.4 mg once weekly vs. placebo	20% relative reduction in MACE in obesity without diabetes; secondary analyses showed fewer HF events; positions semaglutide as cardiometabolic therapy in obesity
SURMOUNT-1 [18]	Obesity/overweight without diabetes	Tirzepatide 5/10/15 mg once weekly vs. placebo	Up to ~ 20% weight loss; marked reductions in waist circumference and hsCRP; establishes dual GIP/GLP-1 agonism as potent weight-loss strategy relevant to obese HFpEF
**HFpEF-obesity trials**			
STEP-HFpEF [24]	Symptomatic HFpEF (LVEF ≥ 45%), BMI ≥ 30 kg/m², without T2D	Semaglutide 2.4 mg once weekly vs. placebo for 52 weeks	Dual primary endpoints: change in KCCQ-CSS and body weight. Improved KCCQ-CSS, 6MWD, NT-proBNP and weight vs. placebo; HF hospitalisations/urgent HF visits numerically fewer with semaglutide (trial not powered for HF events). Gastrointestinal adverse events (mainly nausea and diarrhoea) were more frequent with semaglutide but generally mild–to–moderate and dose-dependent.
STEP-HFpEF DM [25]	Symptomatic HFpEF (LVEF ≥ 45%), BMI ≥ 30 kg/m², with T2D	Semaglutide 2.4 mg once weekly vs. placebo for 52 weeks	Dual primary endpoints: change in KCCQ-CSS and body weight. Similar improvements in KCCQ-CSS, 6MWD and weight to STEP-HFpEF; HF hospitalisation/urgent HF visits numerically fewer with semaglutide (trial not powered for HF events). Gastrointestinal adverse events (mainly nausea and diarrhoea) were more frequent with semaglutide but generally mild–to–moderate and dose-dependent.
SUMMIT [26]	Symptomatic HFpEF (LVEF ≥ 50%), BMI ≥ 30 kg/m²	Tirzepatide (titrated up to 15 mg once weekly) vs. placebo, median follow-up ~ 2 years	Co-primary endpoints CV death or worsening HF (HF hospitalisation or urgent HF visit requiring IV therapy) and KCCQ-CSS. Reduced composite of CV death or worsening HF (driven mainly by fewer worsening HF events); improved KCCQ-CSS, 6MWD, weight, hsCRP and systolic BP vs. placebo. Gastrointestinal adverse events were common but usually mild–to–moderate and manageable with gradual dose escalation.
SUMMIT CMR substudy [27]	SUMMIT participants with analysable CMR at baseline and 52 weeks	Tirzepatide vs. placebo	Reduced LV mass and paracardiac (epicardial + pericardial) fat volume vs. placebo; preserved LVEF and cardiac output; may suggest benefit related to structural remodelling and reduced paracardiac adiposity

Abbreviations: 6MWD, six-minute walk distance; BMI, body-mass index; BP, blood pressure; CMR, cardiovascular magnetic resonance; CVD, cardiovascular disease; DM, diabetes mellitus; GIP, glucose-dependent insulinotropic polypeptide; GLP-1, glucagon-like peptide-1; HF, heart failure; HFpEF, heart failure with preserved ejection fraction; hsCRP, high-sensitivity C-reactive protein; KCCQ-CSS, Kansas City Cardiomyopathy Questionnaire clinical summary score; LVEF, left ventricular ejection fraction; MACE, major adverse cardiovascular events; NT-proBNP, N-terminal pro-B-type natriuretic peptide; T2D, type 2 diabetes. Across these HFpEF trials, safety profiles were consistent with known GLP-1 receptor agonist and dual incretin therapy, with gastrointestinal adverse events the most frequent treatment-related side effects and no new safety signals reported

Semaglutide has been evaluated directly in obesity-related HFpEF in STEP-HFpEF and STEP-HFpEF DM [24, 25]. These trials enrolled adults with symptomatic HFpEF, left ventricular ejection fraction (LVEF) ≥ 45%, body-mass index ≥ 30 kg/m² and elevated natriuretic peptides. STEP-HFpEF included patients with or without type 2 diabetes, whereas STEP-HFpEF DM required type 2 diabetes. In both trials, the dual primary endpoints were change in KCCQ-CSS and percentage change in body weight at 52 weeks; HF outcomes were prespecified secondary or exploratory endpoints, and the studies were not powered for HF event reduction [24, 25]. In each trial, semaglutide 2.4 mg once weekly, added to standard care, improved KCCQ-CSS and six-minute walk distance compared with placebo, and reduced N-terminal pro-B-type natriuretic peptide and body weight. Heart failure (HF) hospitalisations and urgent HF visits were numerically lower with semaglutide, although the studies were not powered to detect differences in the HF outcomes. Gastrointestinal adverse events, mainly nausea and diarrhoea, were more frequent with semaglutide but were usually mild to moderate and rarely led to discontinuation [24, 25].

Tirzepatide, a dual GIP/GLP-1 RA, was tested in SUMMIT, which randomised patients with symptomatic HFpEF. SUMMIT randomised adults with symptomatic HFpEF, LVEF ≥ 50%, body-mass index ≥ 30 kg/m² and elevated natriuretic peptides, with or without type 2 diabetes [26]. Over a median follow-up of around two years, tirzepatide reduced the co-primary composite of cardiovascular death or worsening HF (HF hospitalisation or urgent HF visit requiring intravenous therapy) and improved KCCQ-CSS at 52 weeks, with additional gains in 6-minute walk distance, weight loss, inflammatory markers and systolic blood pressure versus placebo. In the SUMMIT CMR substudy, tirzepatide reduced left ventricular mass and paracardiac (epicardial plus pericardial) fat volume at 52 weeks compared with placebo, with preserved LVEF and cardiac output [27]. Taken together, these findings are consistent with reverse concentric remodelling and reduced pericardial restraint and may reflect associations between weight/fat reduction and favourable cardiac and adipose-tissue remodelling in obesity-related HFpEF.

Although gastrointestinal adverse events predominate in STEP-HFpEF [24, 25] and SUMMIT [26], HFpEF populations warrant closer attention to volume status and renal vulnerability, particularly in those receiving loop diuretics and/or SGLT2 inhibitors, not necessarily included in those trials. During initiation and up-titration, patients should be counselled to maintain oral intake and to report dizziness, symptomatic hypotension, or rapid weight loss suggestive of relative hypovolaemia; diuretic dose may need review if congestion improves while intake falls. A pragmatic approach is to check renal function and electrolytes at baseline and after escalation in higher-risk patients, and to provide “sick-day” advice, temporarily withholding therapy during significant intercurrent illness and restarting once hydration and renal function are stable, often with slower re-titration. These measures align with contemporary cardiovascular weight-management guidance and the tolerability profile reported in HFpEF trials [24, 25]. Clinicians should also counsel patients regarding symptoms of gallbladder disease or pancreatitis, which should prompt interruption and evaluation.

Overall, the HFpEF–obesity trials to date establish improvements in symptoms, functional capacity and congestion, with more robust evidence for reduction in HF events currently available only for tirzepatide in SUMMIT, while effects on mortality, long-term durability of benefit and generalisability beyond obese HFpEF phenotypes remain unproven [24–26].

### Multiorgan Incretin Trials Beyond the Heart

**Fig. 1:**
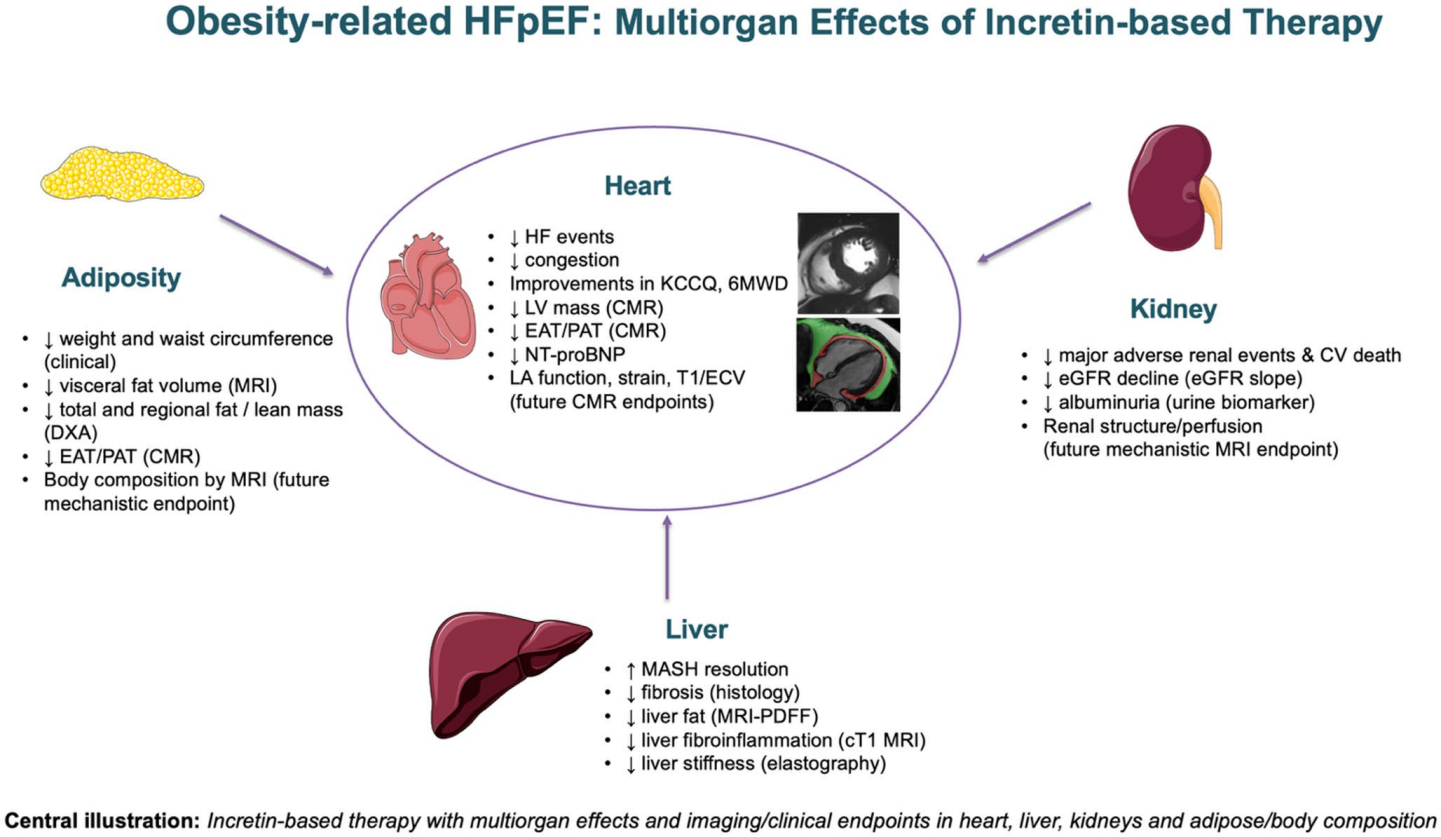
Central illustration: Incretin-based therapy with multiorgan effects and imaging/clinical endpoints in heart, liver, kidneys and adipose/body composition. **Core clinical endpoints** for obesity-related HFpEF trials include HF events and congestion, major renal events and cardiovascular death, symptoms/quality of life (KCCQ-CSS), functional capacity (6MWD), body weight and central adiposity, MASH resolution and liver fibrosis, and renal function (eGFR and albuminuria). **Core imaging endpoints** include LV mass, visceral and epicardial/pericardial fat volumes, and liver fat and fibroinflammation (MRI-PDFF and cT1). **Exploratory endpoints** shown in the panels (e.g. LA function, myocardial strain, T1/ECV, whole-body/body-composition MRI, liver stiffness and renal structural/perfusion imaging) are primarily used for phenotyping and mechanistic substudies rather than as primary trial endpoints. Arrow symbols (↑/↓) indicate the direction of change from baseline to follow-up where trial results are available. Bullets without arrows denote exploratory measures for which treatment effects are not summarised here. Abbreviations: 6MWD, six-minute walk distance; CMR, cardiovascular magnetic resonance; CV, cardiovascular; DXA, dual-energy X-ray absorptiometry; EAT, epicardial adipose tissue; ECV, extracellular volume; eGFR, estimated glomerular filtration rate; GLP-1, glucagon-like peptide-1; GIP, glucose-dependent insulinotropic polypeptide; HF, heart failure; HFpEF, heart failure with preserved ejection fraction; KCCQ, Kansas City Cardiomyopathy Questionnaire; LA, left atrium; LV, left ventricle; MASH, metabolic dysfunction–associated steatohepatitis; MRI-PDFF, MRI-derived proton density fat fraction; NT-proBNP, N-terminal pro-B-type natriuretic peptide; PAT, pericardial adipose tissue; RA, receptor agonist; cT1, iron-corrected T1.

Non-cardiac incretin trials complement the HFpEF studies and reinforce the view of obesity-related HFpEF as part of a broader CRHM syndrome *(*Fig. 1*).* Selected non-cardiac incretin trials with prespecified multiorgan endpoints – including liver histology or liver fat, kidney outcomes and body-composition measures – are summarised in Table 2, focusing on randomised phase 2–3 studies of GLP-1 RA and GIP/GLP-1 agonist therapy in obesity, MASLD/MASH (previously known as NASLD/NASH) and chronic kidney disease.

**Table 2:** Non-cardiac incretin trials with liver, kidney and adipose endpoints

Trial	Population	Intervention vs. placebo	Key findings
Semaglutide NASH phase 2 [19]	Adults with biopsy-confirmed MASH (previously termed NASH) and fibrosis	Semaglutide (once daily) vs. placebo	Higher rates of MASH resolution without worsening of fibrosis; substantial weight loss; reductions in liver fat and liver enzymes, supporting histology and liver fat as endpoints
Semaglutide NAFLD MRI-PDFF trial [28]	Adults with MASLD (previously termed NAFLD)	Semaglutide vs. placebo	Reduction in liver fat measured by MRI-PDFF, often without major change in stiffness; demonstrates MRI-PDFF as a sensitive quantitative liver-fat endpoint
SYNERGY-NASH (tirzepatide) [29]	Adults with metabolic MASH (previously termed NASH) and stage 2–3 fibrosis	Tirzepatide vs. placebo	High rates of MASH resolution without worsening of fibrosis; improvements in non-invasive fibrosis markers; supports dual incretin agonism as a liver-targeted therapy
FLOW (Semaglutide kidney outcomes) [20]	Type 2 diabetes with chronic kidney disease	Semaglutide 1.0 mg once weekly vs. placebo	Reduced composite of major adverse kidney events and cardiovascular death; slower decline in eGFR and lower albuminuria; supports eGFR slope and albuminuria as renal endpoints within hierarchical multiorgan endpoint frameworks
ESSENCE (semaglutide in MASH) [30]	Adults with biopsy-confirmed MASH and stage 2–3 fibrosis	Semaglutide 2.4 mg once weekly vs. placebo	Higher rates of MASH resolution and fibrosis improvement versus placebo, with substantial weight loss and metabolic benefits; reinforces semaglutide as a liver- and metabolically directed therapy suitable for multiorgan endpoint trials

Terminology for metabolic liver disease has been updated from NAFLD/NASH to MASLD/MASH but legacy terms NAFLD and NASH are retained only where they form part of original trial names. abbreviations: CKD, chronic kidney disease; EGFR, estimated glomerular filtration rate; MASLD, metabolic dysfunction–associated steatotic liver disease; MASH, metabolic dysfunction–associated steatohepatitis; MRI-PDFF, mri-derived proton density fat fraction

In the liver, the phase 2 semaglutide trial in biopsy-confirmed non-alcoholic steatohepatitis (NASH, currently known as MASH) demonstrated higher rates of steatohepatitis resolution than placebo, along with substantial weight loss and reductions in liver enzymes and liver fat content [19]. MRI-based semaglutide trials in MASLD have shown reductions in liver fat measured by MRI–proton density fat fraction (MRI-PDFF), even when liver stiffness is unchanged, supporting MRI-PDFF as a quantitative liver-fat endpoint [28]. The phase 3 ESSENCE trial extended these findings by showing that once-weekly semaglutide 2.4 mg achieved higher rates of MASH resolution and fibrosis improvement than placebo in patients with stage 2–3 fibrosis, alongside substantial weight loss and metabolic benefits [30]. On the basis of ESSENCE, semaglutide has now received regulatory approval for MASH with fibrosis in some regions, providing a precedent for liver-directed indications in MASLD/MASH [30]. In the United States, semaglutide 2.4 mg (Wegovy) received FDA accelerated approval for adults with non-cirrhotic MASH with moderate-to-advanced fibrosis, with continued approval contingent on confirmatory evidence of clinical benefit [31]. In the European Union, the Committee for Medicinal Products for Human Use at the European Medicines Agency (EMA) has recommended conditional marketing authorisation for semaglutide (Kayshild) for non-cirrhotic MASH with fibrosis, reflecting an expedited pathway requiring additional post-authorisation data [32]. The phase 2 trial of tirzepatide in metabolic steatohepatitis with stage 2–3 fibrosis (SYNERGY-NASH) has also reported high rates of steatohepatitis resolution without worsening of fibrosis, supporting its recent progression to Phase 3. Together, these results support the application of dual incretin agonism as a liver-targeted therapy [29].

In the kidney, the FLOW trial established once-weekly semaglutide as the first GLP-1 RA with dedicated kidney outcome data, reducing a composite of major kidney events and cardiovascular death and slowing the decline in estimated glomerular filtration rate in patients with type 2 diabetes and chronic kidney disease [20]. These findings are highly relevant to obesity-related HFpEF, where albuminuric nephropathy and chronic kidney disease are common and contribute to congestion, neurohumoral activation and adverse prognosis.

Body-composition substudies from obesity programmes such as STEP-1 and SURMOUNT-1 show that semaglutide and tirzepatide reduce visceral fat and waist circumference to a greater extent than would be expected with parallel improvements in inflammatory and metabolic markers [17, 18]. In SUMMIT CMR, reductions in paracardiac (epicardial and pericardial) fat were documented directly [27].

Collectively, these multiorgan data support the concept that incretin-based therapies act across the liver, kidney and adipose tissues, providing a rationale for incorporating multiorgan imaging and biomarker endpoints in obesity-related HFpEF trials.

## Multiorgan imaging endpoints in obesity-related HFpEF

As obesity-related HFpEF is increasingly viewed as a multiorgan syndrome, imaging endpoints should extend beyond conventional measures of left ventricular structure and function [1, 4, 5], *(*Fig. 1*).* In this context, multiorgan imaging serves not only for descriptive phenotyping but also for mechanistic insight and as potential surrogate endpoints for therapeutic response across cardiac, hepatic, adipose and renal systems. Echocardiography remains the first-line imaging modality for HFpEF diagnosis alongside clinical assessment, but image quality is often suboptimal in individuals with a high body mass index, and assessment is largely confined to cardiac structure and haemodynamics rather than extracardiac organ involvement [3]. From a cardiovascular perspective, core cardiac imaging endpoints in obese HFpEF include LV volumes and mass, ejection fraction, left atrial volumes and phasic function, indices of diastolic function and global longitudinal strain, together with quantitative assessment of EAT and PAT volumes, which are commonly used as surrogate markers of haemodynamic load, congestion risk and symptom burden.

Cardiovascular magnetic resonance (CMR) offers highly reproducible quantification of ventricular volumes, mass and ejection fraction and is well suited to mechanistic and phase 2 studies, with established multicentre reproducibility and standardised protocols that make it a scalable endpoint for larger trials [3]. Of particular relevance to obesity-related HFpEF, CMR can directly quantify epicardial and pericardial adipose tissue volumes and, in some centres, myocardial triglyceride content, allowing integrated assessment of cardiac structure, function and ectopic fat in a single examination [8, 27]. For phase 2/2b mechanistic trials in obese HFpEF, a pragmatic core CMR set would include LV volumes and mass, left atrial volumes and phasic function, and global longitudinal strain, where available, and quantitative EAT/PAT volumes, with the direction of benefit expected to be reductions in LV mass, LA size, EAT/PAT and improved strain, although minimal clinically important changes for these measures in obese HFpEF have not yet been established (no universally accepted thresholds).

For extracardiac organs, MRI–proton density fat fraction (MRI-PDFF) provides a quantitative measure of hepatic steatosis, is sensitive to change over time, and is well-suited as a secondary endpoint in incretin trials that include HFpEF patients [19, 28]. Multiparametric liver MRI (such as iron-corrected T1 [cT1]) provides non-invasive surrogates of hepatic fibroinflammation and can be acquired in the same session as CMR. Abdominal MRI or CT scan can quantify visceral and subcutaneous fat depots, while dual-energy X-ray absorptiometry provides complementary information on total and regional fat and lean mass; abdominal MRI has the advantage of avoiding both contrast and ionising radiation, whilst also being able to be acquired in the same scanning session as other organs [28, 29]. Taken together, for phase 2/2b mechanistic trials in obese HFpEF, a practical multiorgan MRI endpoint set would include liver fat burden assessed by MRI-PDFF, a liver fibroinflammation surrogate such as iron-corrected T1 (cT1), and a measure of central adiposity (for example, abdominal MRI–derived visceral fat volume or DXA-derived regional fat mass). These endpoints map directly onto the weight-dependent, ectopic-fat and weight-independent pathways outlined above and are primarily interpreted as directional changes, as universally accepted minimal clinically important thresholds for these measures in obese HFpEF have not yet been defined.

Renal imaging is less established in HFpEF trials. Routine renal endpoints – estimated glomerular filtration rate, creatinine and albuminuria – remain fundamental and are likely to be the primary renal measures in most studies [20]. More advanced renal MRI techniques (for example, perfusion or diffusion imaging) can characterise renal structure and microcirculation more directly but are likely to be confined to mechanistic substudies. One example is the REMODEL trial in type 2 diabetes and chronic kidney disease, which uses multiparametric renal MRI together with kidney biopsies and biomarkers to investigate the renal mechanisms of semaglutide, with full results still awaited [33].

Experience from multiorgan cardiometabolic imaging programmes suggests that harmonised “heart–liver–fat” MRI protocols are feasible. A single session can combine cine CMR, myocardial T1 and T2 mapping, epicardial and pericardial fat quantification, MRI-PDFF and cT1 [8, 27–29]. For multicentre phase 2/2b studies, reproducibility can be optimised by using standardised acquisition protocols across vendors, central training and accreditation, phantom-based calibration where available, and blinded core-laboratory analysis for key endpoints such as LV mass, EAT/PAT volumes, MRI-PDFF and liver fibroinflammation surrogates [34]. Feasibility in severely obese HFpEF populations can be enhanced by limiting scan time, patient-tailored positioning, and adopting a tiered imaging strategy in which all participants undergo core sequences while more intensive multiorgan protocols are reserved for predefined substudies to minimise missing data and participant burden [35, 36].

## Current position in guidelines and future directions

Obesity-related HFpEF is increasingly recognised as a distinct phenotype, yet current HF guidelines have not incorporated GLP-1 receptor agonists or dual incretin agonists as recommended HF therapies, reflecting the evolving evidence base [3–5]. The 2023 European Society of Cardiology Focused Update strengthened recommendations for SGLT2 inhibitors in HFpEF (Class I, Level A), supporting SGLT2 inhibition as foundational disease-modifying therapy across the HFpEF spectrum, alongside diuretics for symptomatic congestion relief and systematic optimisation of key comorbidities such as hypertension, atrial fibrillation and coronary artery disease [37]. Within this framework, incretin-based agents are best positioned as adjunctive, phenotype-targeted therapies in patients with obesity-related HFpEF, implemented primarily to address upstream drivers such as adiposity, insulin resistance, systemic inflammation and multi-organ dysfunction rather than as core HF therapies per se [37]. This positioning is consistent with contemporary American College of Cardiology consensus guidance on medical weight management in cardiovascular practice, which outlines multidisciplinary pathways for patient selection, initiation and dose escalation, monitoring, and adverse-event mitigation — issues that are particularly relevant in HF populations (for example volume status, renal function, gastrointestinal intolerance and preservation of lean mass during weight loss) [38].

Regulatory indications for incretin-based treatments now include chronic weight management in people with obesity and, in some regions, cardiovascular risk reduction in obesity without diabetes, with additional evidence supporting their use in chronic kidney disease and steatohepatitis [16–20, 29, 30]. STEP-HFpEF, STEP-HFpEF DM and SUMMIT provide proof of concept that semaglutide and tirzepatide can improve symptoms and exercise capacity in obesity-related HFpEF and, in the case of tirzepatide, reduce HF events and may favourably remodel the heart and paracardiac fat [25–27]. However, longer-term HF-specific data on mortality, durability of benefit, adherence and cost-effectiveness are still needed before guideline recommendations are likely to change [3, 5].

## Key implementation considerations

The durability of benefit, particularly after treatment discontinuation, has not been established, and real-world tolerability in symptomatic HFpEF may be less favourable than in general obesity cohorts, given older age, multimorbidity, fluid overload and polypharmacy. Mechanistic attribution also remains unresolved: improvements in symptoms and exercise capacity may reflect differing contributions from increased physical activity/fitness, decongestion, haemodynamic unloading, and true reverse remodelling. In addition, apparent treatment effects may be modified or confounded by concurrent optimisation of background HF and comorbidity therapies (including diuretic adjustment and initiation of disease-modifying agents). Finally, the translational impact will be shaped by cost-effectiveness and equitable access, with substantial variation in reimbursement and service delivery across health systems.

Future phase 2b–3 trials should deliberately target the obesity-related HFpEF phenotype, combining criteria for HFpEF, obesity and central adiposity. A practical enrichment approach would combine BMI ≥ 30 kg/m² with a sex-specific waist circumference threshold and at least one marker of ectopic or central fat burden (e.g. imaging-derived visceral fat volume, elevated epicardial or paracardiac fat, or non-invasive evidence of MASLD) to select patients in whom adiposity-driven multiorgan dysfunction is most likely. Such enrichment would be expected to increase the signal for changes in LV mass, left atrial function, EAT/PAT burden, liver fat and fibroinflammation, and visceral adiposity, thereby improving the efficiency of phase 2/2b mechanistic studies. These enrichment criteria and mechanistic hypotheses are applicable to the obese HFpEF phenotype with central and ectopic adiposity, as in STEP-HFpEF and SUMMIT, and should not be extrapolated to lean or non-obese HFpEF phenotypes. A typical design would randomise such patients to GLP-1 RA or dual incretin therapy versus placebo on top of guideline-directed HF treatment, with a cardiovascular-driven primary endpoint [5, 24–26]. Prespecified imaging substudies incorporating harmonised “heart–liver–fat” MRI to quantify changes in left ventricular mass, left atrial function, epicardial and pericardial fat, liver fat, fibroinflammation and abdominal visceral fat, alongside renal endpoints and biomarker panels, may be particularly informative [8, 19, 20, 27–29]. These studies will be necessary to define the role of incretin-based, weight-directed therapies in routine care and to establish a broader framework for evaluating other multiorgan interventions in obesity-related HFpEF. Combining these imaging markers with clinical endpoints would help establish whether multiorgan imaging changes can serve as surrogate or supportive endpoints for clinical benefit.

## Conclusion

Obesity-related HFpEF should be reframed as an adiposity-driven, multiorgan cardio–renal–hepatic–metabolic syndrome that is not optimally addressed by conventional HF therapies. GLP-1 receptor agonist and dual incretin agonist therapies produce substantial weight and visceral fat loss, improve metabolic and inflammatory profiles, and exert favourable multiorgan effects, and these changes may be accompanied by improvements in symptoms, exercise capacity, and, in selected trials, fewer HF events.

Although not yet guideline-endorsed as HF drugs, incretin-based, weight-directed treatments are emerging as key components of the management of obesity-related HFpEF. Future randomised trials should integrate multiorgan cardiac, hepatic, adipose and renal imaging with clinical endpoints to help define the role of incretin therapies in this multiorgan disease phenotype.

## Data Availability

No datasets were generated or analysed during the current study.
